# Editorial: Turn-Taking in Human Communicative Interaction

**DOI:** 10.3389/fpsyg.2015.01919

**Published:** 2015-12-21

**Authors:** Judith Holler, Kobin H. Kendrick, Marisa Casillas, Stephen C. Levinson

**Affiliations:** Max Planck Institute for PsycholinguisticsNijmegen, Netherlands

**Keywords:** turn-taking, turn-timing, turn transitions, conversation, social interaction, language processing, prediction, projection

One intriguing feature of the human communication system is the interactional infrastructure it builds on. In both dyadic and multi-person interactions, conversation is highly structured and organized according to set principles (Sacks et al., 1974). Human adult interaction is characterized by a mechanism of exchange based on alternating (and relatively short) bursts of information. In the majority of cases, only one person tends to speak at a time and each contribution usually receives a response. What is remarkable is the precise timing of these sequential contributions, resulting in gaps between speaking turns averaging around just 200 ms (Stivers et al., 2009). From psycholinguistic experiments, we know that the time it takes to produce even simple one-word-utterances (min. 600 ms, Indefrey and Levelt, 2004) by far exceeds this average gap duration, hinting at the complexity of the cognitive processes that must be involved (Levinson, 2013).

While the behavioral principles governing turn-taking in interaction have been researched for some decades—primarily by scholars of conversation analysis—the cognitive underpinnings of the human turn-taking system have long remained elusive. Recently, psycholinguists have begun to explore the cognitive and neural processes that allow us to deal effectively with the immensely complex task of taking turns on time. Amongst other things, this has highlighted the anticipatory, predictive processes that must be at work, as well as the different layers of processing allowing production planning and comprehension to take place simultaneously (de Ruiter et al., 2006; Magyari and de Ruiter; Bögels et al., 2015). These insights mesh well with the conversation analytic literature that has illuminated the interactional environments in which individual turns are embedded: their sequential organization and the use of conventionalized linguistic constructions allow for the projection of upcoming talk, as well as for the recognition of points of possible completions in the turn which make transition to the next speaker relevant (Sacks et al., 1974; Ford and Thompson, 1996; Schegloff, 2007). The articles in this Research Topic bring together these as yet largely independent lines of research to elucidate our understanding of turn-taking from multiple perspectives and aim to foster future synergies.

In addition to exploring the adult psycholinguistic machinery and its workings, researchers have begun to wonder how and when the required cognitive and social processes mature in children, as well as how they compare to those in other species. Levinson (2006) proposed that human beings are inherently social and interactive in orientation. He argues that an “interaction engine” may lie at the heart of children's early predisposition for turn-taking. Likewise, this particular human capacity might explain the strong cultural universals in the structure of human interaction as well as the striking commonalities and differences in communication systems brought about by the course of evolution.

The present Research Topic provides a collection of experimental and observational empirical studies using qualitative and quantitative approaches, complemented by articles offering reviews, opinions, and models. They aim to inform the reader about the most recent advances in our endeavor of unraveling the workings of the human turn-taking system in communicative interaction. The contributions are organized into six sections: (1) Foundations of turn-taking, (2) Signals and mechanisms for prediction and timing, (3) Planning next turns in conversation, (4) Effects of context and function on timing, (5) Turn-taking in signed languages, and (6) Development of turn-taking skills.

## Foundations of turn-taking

The articles in this section outline models of human turn-taking, specify the interaction of the various psycholinguistic processes that underlie our ability to take conversational turns on time, and test the applicability of human turn-taking models to non-human animal species. Levinson and Torreira review behavioral and cognitive findings specifying the parameters of the processes underlying the human turn-taking system. This empirical evidence is synthesized into a model claiming that intention ascription and response planning begin as early as possible during the incoming turn, running through all the serial stages of speech production à la Levelt (1989) before the response is launched, triggered by turn-final cues. Garrod and Pickering propose a model that specifies two processes. The first is based on the entrainment of brain oscillations that allow listeners to predict when the incoming turn will end. The second is constrained by the first and based on covert imitation, allowing listeners to determine the intention conveyed by the incoming turn. The final article in this section addresses the phylogenetic development of turn-taking skills. Henry et al. look at the European Starling's turn-taking behavior, finding evidence for both temporal and structural regularities, the influence of the immediate as well as the wider social context in which turns are produced, and of emitter-specific factors influencing the behavior—thus pointing toward strong similarities with some of the features shaping turn-taking in humans. In addition, they provide comparisons with other starling species, leading the authors to argue for turn-taking behavior having co-evolved in close interdependency with social structure.

The empirical studies collected in the rest of this Research Topic support various components of these proposed turn-taking models while in places being at odds with some of the claims made. As much as the current volume is a summary of the state-of-the-art in the field, it also aims to stimulate future research that will help us piece together the parts of the remarkable puzzle that human turn-taking poses.

## Signals and mechanisms for prediction and timing

One of the central debates on the cognitive processes involved in turn-taking focuses on the role played by prediction. Part of this debate is the issue of which kinds of cues adults may use for predicting the end of turns, allowing them to come in on time. The article by Riest et al. further advances this debate by testing, in three offline experiments, the relative contribution of syntactic, and semantic information to turn-end anticipation. It shows that, while both types of information are essential, adults rely predominantly on the latter. The article by Holler and Kendrick builds on this work by using eye-tracking technology to investigate the responses of observers directly immersed in a conversational setting. The data show that observers' eye movements toward next speakers are not random but guided by points of possible completion in current turns, thus revealing interactants' sensitivity and orientation toward the semantic, syntactic, prosodic, and pragmatic information that becomes available as turns unfold. The article by Hiroko zooms into the projective power of specific lexicogrammatical particles in Japanese (*wa, mo*, and *tte*). These become available to listeners as turns unfold in conversation and often allow next speakers to predict the content of ongoing turns. Himbert et al. throw light on yet another source of information that speakers in interaction may use for timing their turns: their analysis demonstrates that interlocutors adapt their turn-taking rhythms to one another, which they argue is facilitated by the alignment of semantic and syntactic processes.

## Planning next turns in conversation

The contributions in this section explore some of the cognitive processes involved in preparing next turns in conversation. Applying a cutting edge statistical approach (“random forests”) to data from a large conversational corpus, Roberts et al. explore the value of both psycholinguistic factors (e.g., word frequency and syntactic complexity) and conversational structures (e.g., the sequential relationships between turns) as explanatory factors when modeling the timing of turns in conversation. Their results show that both sets of factors significantly contribute to explaining variation in turn timing. Torreira et al. study pre-answer in-breaths in a dialogue setting using insights from acoustic and inductive plethysmography recordings. They demonstrate that the occurrence of an in-breath is dependent on the length of an answer, suggesting that answers are planned prior to these in-breaths. Since the pre-answer in-breaths in their data were launched close to the end of question turns, the data provide evidence for the concurrence of comprehension and next utterance planning.

## Effects of context and function on timing

Three articles investigate the interplay of turn-taking rules with other principles shaping human behavior in specific conversational contexts. Kendrick shows that turns dealing with problems of speaking, hearing, and understanding (i.e., other-initiations of repair) are governed by different timing principles and can thus break the common pattern of minimal gaps between turns. As the analysis reveals, the longer gaps characteristic of repair sequences tend to be used by participants as opportunities to either allow the producer of the trouble source to resolve the issue before repair is initiated, to allow themselves to resolve their problems in understanding before initiating repair, or to signal problems in understanding through visual displays (e.g., eyebrow raise) before initiating repair verbally. The article by Gardner and Mushin provides evidence from Garrwa, an indigenous Australian language, for turns that are followed by substantially longer gaps than one would ordinarily expect based on prior work on English conversations. In these cases, however, it is not repair that drives the longer turn transition times; the environment in which they occur is slow-paced conversation, appearing to reduce the pressure for gap minimization. Stevanovic and Peräkylä discuss perspectives on the intersection of two different systems of temporal organization, that of turns at talk and that of emotional reciprocity—the former favoring sequential organization, the latter affording simultaneity and immediate uptake through emotional contagion and mimicry.

## Turn-taking in signed languages

The research presented in this section investigates the principles of turn-taking and sequence organization in signed languages where communication is constrained to the visual modality. De Vos et al. analyze the timing of turns in Sign Language of the Netherlands (NGT), showing that the timing of turns in signed conversation looks remarkably similar to that of spoken interaction (i.e., with minimal gaps and minimal overlaps) when considering not simply onset and offset of manual movements but individual movement phases (preparations, strokes, retractions). Girard-Groeber examines turn-taking principles in multi-party conversations in Swiss German Sign Language (DSGS), focusing on the occurrence of overlaps. She, too, finds striking similarities with spoken interactions: the examples provided illustrate a strong orientation to the “one at a time” principle, an orientation of participants toward points of possible completion in the sign stream, and a set of principles that appear to determine deviations from this rule (such as repair initiations or strong disagreements). Manrique and Enfield focus on a particular type of turn transition environment—other-initiated-repair—in Argentine Sign Language (LSA), thus complementing Kendrick's work on repair in spoken interaction (this volume). However, their focus is on how repair is elicited in visual question-answer sequences rather than on the timing of turns in the repair environment, revealing the frequent use of a visual display form termed the “freeze-look.” Next to clearly unique features, the three articles point toward some striking similarities regarding the timing and organization of turns in spoken and signed languages.

## Development of turn-taking skills

Convergent findings regarding principles governing turn-taking across languages in different modalities hint at the possibility of a shared cognitive infrastructure underlying all human communicative interaction. This cognitive infrastructure may also account for the ease with which young children appear to acquire the necessary skills to interact with others. The contributions included in this section focus on the acquisition of turn-taking in very young infants and in children as they start to master spoken language. The first two articles suggest that temporal turn-taking skills are learned early on in infancy. Gratier et al. demonstrate that already at 8–21 weeks babies are active participants in, as well as initiators of, turn-taking sequences, but also that at this early stage of development mothers play a core role in the timing of turns by adapting their behavior to the infant. Hilbrink et al. provide a longitudinal study showing that turn-timing skills continue to develop continuously from 3 to 18 months, with some regressive slowing down as language comprehension kicks in around the “9 month revolution” (Tomasello, 2008). Clark and Lindsey provide a case study of one child's (1;4-3;5 years) verbal and gestural responses to questions. The pattern they find nicely fits with the temporal slowing down in vocal turn-timing caused by the challenge of having to master language—while verbal responses often occurred with long delays, the child frequently produced gestural responses preceding speech. The following two articles examine children's use of linguistic cues for anticipating upcoming next turns when observing dyadic conversations. Keitel and Daum find that three but not 1 year olds are able to make use of intonational cues for predicting upcoming next turns. In line with this, Lammertink et al. find that 2 year olds make use of prosodic cues for predicting upcoming next turns, but that they make use of lexicosyntactic cues, too, even weighing these more strongly—just like adults do.

## Funding

The authors were supported through the Max Planck Gesellschaft and European Research Council (Advanced grant #269484 INTERACT awarded to SCL) during the preparation of the editorial and the research topic as a whole.

### Conflict of interest statement

The authors declare that the research was conducted in the absence of any commercial or financial relationships that could be construed as a potential conflict of interest.
